# Predicting the probability of H3K4me3 occupation at a base pair from the genome sequence context

**DOI:** 10.1093/bioinformatics/btt126

**Published:** 2013-03-19

**Authors:** Misook Ha, Soondo Hong, Wen-Hsiung Li

**Affiliations:** ^1^Department of Ecology and Evolution, University of Chicago, 1101 East 57th Street, Chicago, IL 60637, USA, ^2^Future IT Research Center, Samsung Advanced Institute of Technology, Samsung Electronics Corporation, Yongin-City, Gyeonggi 446-712, ^3^Systems Engineering Team, Samsung Display Corporation, Asan-City, Chungnam 336-741, South Korea and ^4^Biodiversity Research Center, Academia Sinica, Taipei 115, Taiwan

## Abstract

**Motivation:** Histone modifications regulate chromatin structure and gene expression. Although nucleosome formation is known to be affected by primary DNA sequence composition, no sequence signature has been identified for histone modifications. It is known that dense H3K4me3 nucleosome sites are accompanied by a low density of other nucleosomes and are associated with gene activation. This observation suggests a different sequence composition of H3K4me3 from other nucleosomes.

**Approach:** To understand the relationship between genome sequence and chromatin structure, we studied DNA sequences at histone modification sites in various human cell types. We found sequence specificity for H3K4me3, but not for other histone modifications. Using the sequence specificities of H3 and H3K4me3 nucleosomes, we developed a model that computes the probability of H3K4me3 occupation at each base pair from the genome sequence context.

**Results:** A comparison of our predictions with experimental data suggests a high performance of our method, revealing a strong association between H3K4me3 and specific genomic DNA context. The high probability of H3K4me3 occupation occurs at transcription start and termination sites, exon boundaries and binding sites of transcription regulators involved in chromatin modification activities, including histone acetylases and enhancer- and insulator-associated factors. Thus, the human genome sequence contains signatures for chromatin modifications essential for gene regulation and development. Our method may be applied to find new sequence elements functioning by chromatin modulation.

**Availability:** Software and supplementary data are available at *Bioinformatics* online.

**Contact:** misook.ha@samsung.com or wli@uchicago.edu

**Supplementary information:** Supplementary data are available at *Bioinformatics* online.

## 1 INTRODUCTION

Chromatin remodeling mediated by histone modifications is an important mechanism for specific gene expression (Ha *et al.*, 2011). However, the relationship between genome sequence and chromatin remodeling is not well understood. Although DNA sequence preferences for nucleosome formation have been known since the mid 1980s (Drew and Travers, 1985; Struhl, 1985; Yuan *et al.*, 2005), the role of histone–DNA interaction in nucleosome formation is subjected to debate (Kaplan *et al.*, 2009; Zhang *et al.*, 2009). The interplay between transcription factors (TFs) and adenosine triphosphate-dependent chromatin modulating factors in regulating histone modifications implies that histone modifications do not follow the sequence preferences of the general H3 nucleosomes or the other nucleosomes that are not trimethylated at H3K4 (Berger, 2007; Jenuwein and Allis, 2001; Matthews *et al.*, 2007). Indeed, the H3K4me3 nucleosome is preferentially enriched in the genomic regions showing a low density of other nucleosomes and marks an open chromatin region. The advance of DNA sequencing technology with immunoprecipitated DNA segment associated with specific histone modifications allows us to examine the DNA sequence compositions specific to histone modifications in an unbiased way. Therefore, we extracted DNA sequences of nucleosomes carrying individual histone modifications from ChIP-seq data (Barski *et al.*, 2007; Cui *et al.*, 2009; Hawkins *et al.*, 2010; Lister *et al.*, 2009; Wang *et al.*, 2008, 2009b) and identified the features of sequences bound to histone modifications in ChIP-seq experiments. Using the identified sequence specificities of H3K4me3 and H3 nucleosomes, we developed a model to compute the probabilities of H3K4me3 and H3 nucleosome occupation from the genome sequence context. H3K4me3 ChIP-seq data in various human cells indicate that the loci predicted by our model to have a high probability of the H3K4me3 sequence signature are preferentially occupied by H3K4me3 nucleosome. Our study provides a method for investigating the DNA sequence features of chromatin structure. Furthermore, our analyses show that the human genome sequence contains signals for chromatin remodeling at epigenetic regulatory elements.

## 2 METHODS

### 2.1 ChIP-seq data sources

The ChIP-seq data of H3 nucleosomes in two different conditions of human CD4 + T cells were obtained from Schones *et al.* (2008). The ChIP-seq data of CTCF, H3K4me1, H3K4me2, H3K4me3, H3K27me3, RNA Pol II in human CD4 + T cells, CD133 + and CD36 + cells were obtained from Barski *et al.* (2007) and Cui *et al.* (2009). Methyl-bisulfite sequencing data and ChIP-seq data of various histone methylations in human embryonic stem cells (HESC) are from Lister *et al.* (2009) and Hawkins *et al.* (2010). The ChIP-seq data of histone acetylases, deacetylases and acetylations are from Wang *et al.* (2008, 2009b).

### 2.2 Identification of sequence specificities of nucleosome modifications

The 30–50 bp sequences from the ChIP-seq data are mapped to the February 2009 human reference sequence (GRCh37/hg19) by perfect and unique match without allowing any mismatch or gap. To recover nucleosomal DNA fragments, each read was extended toward its 3′-end by 151 bp; the 30–50 bp reads are from the ends of both strands in nucleosome DNAs. The possible range of the ends of nucleosome-bound DNAs may be wider than that of micrococal nuclease-treated DNAs. Therefore, we consider 151 bp nucleosome-bound DNA regions, and from these regions, we estimate the frequencies of monomer, 2, 3, 4, 5 and 6mer sequences for each histone modification and each cell type. The sequence frequencies are normalized by the sequence composition in the reference genome sequence (hg19/GRCh37) to account for bias because of genome sequence composition. In this way, we estimate the enrichment of nucleosomes at every sequence composition.

### 2.3 A probabilistic model of H3 and H3K4me3 nucleosome occupation in a genome

The occurrence of an H3 or H3K4me3 nucleosome at a genomic site can be affected by adjacent sequences or adjacent nucleosomes. Across a genome, we calculate the probability that a nucleosome (H3 or H3K4me3) locates at a locus by using the modified fifth order hidden Markov model (HMM) (Rabiner, 1989). This model considers possible arrangements in view of the competition with adjacent nucleosomes in evaluating the forward and backward status and sequence composition of the147 bp sequence.

#### 2.3.1 Forward procedure

Using the forward procedure, we calculate the probability of each status at the *ith* position considering the genome sequence from the first bp *S*_1_ to *S_i_*.

*M_i_*: The *ith* base pair in the DNA sequence of an H3K4me3 nucleosome.

*N_i_*: The *ith* base pair the DNA sequence of an H3 nucleosome.


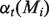
: The probability that the sequence from *S*_1_ to *S_t_* is observed, and *S_t_* is the *ith* bp of the DNA sequence of an H3K4me3 nucleosome.


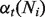
: The probability that the sequence from *S*_1_ to *S_t_* is observed, and *S_t_* is the *ith* bp of the DNA sequence of an H3 nucleosome.


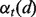
: The probability that the sequence from *S*_1_ to *S_t_* is observed, and *S_t_* is not bound by any H3 or H3K4me3 nucleosome.

**(1) Initialization**



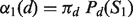



*d*: Depletion of all nucleosomes


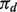
: Proportion of nucleosome-depleted regions in the genome


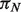
: Proportion of nucleosome-bound regions in the genome


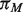
: Proportion of H3K4me3 nucleosome-bound regions in the genome

**(2) Induction**



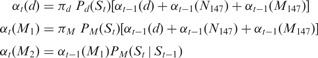


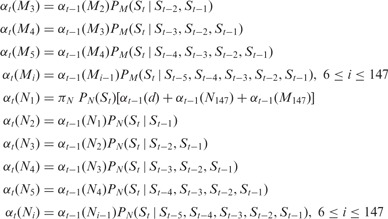



**(3) Termination**


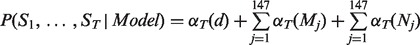
, where *T* is the total length of the genome.

#### 2.3.2 Backward procedure

Using the backward procedure, we calculate the probability of observing the sequence, from 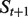 to the end of the genome with an H3K4me3 nucleosome at 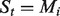.


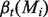
: Probability of the sequence from 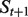 to the end of the genome when 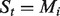.


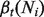
: Probability of the sequence from 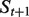 to the end of the genome when 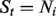.


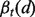
: Probability of the sequence from 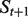 to the end of the genome when 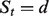.

**(1) Initialization**



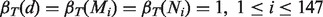



**(2) Induction**



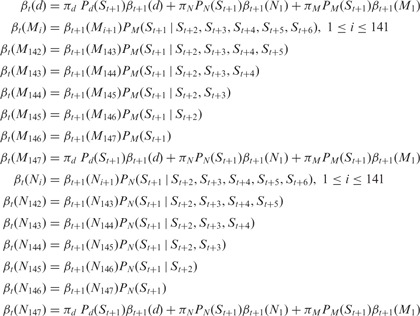



#### 2.3.3 Integration of backward and forward probabilities

The probability that the *tth* bp is at *M_i_* is normalized by the sum of all configurations in our model.



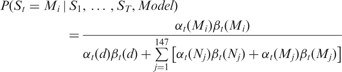



#### 2.3.4 The H3K4me3 sequence signature (the probability of H3K4me3 occupation based on DNA primary sequence)

The probability that a base pair position can potentially be covered by an H3K4me3 can be calculated by summing the probabilities from *M*_1_ to *M*_147_. 
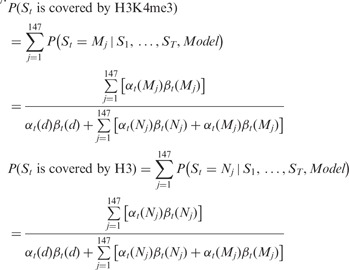



### 2.4 *In vivo* coverage of modified nucleosomes at a base pair

The ChIP-seq reads obtained from published data were mapped to the human genome sequence (hg19). Each 30–50 bp read perfectly matching the genome sequence only once was extended to 151 bp from the 5′-end of the read because the immunoprecipitated DNA fragments are 150 bp in length. The coverage of each histone modification at a base pair position was estimated by the number of extended reads covering that position. As the coverage is dependent on the depth of ChIP-seq, we use correlation coefficients that are standardized by standard deviations. The comparisons were made in the mappable genomic regions.

### 2.5 Comparison of the probabilistic H3K4me3 occupation map with *in vivo* data

To validate the predicted H3K4me3 occupation map, we calculate the correlation coefficient between the probability of H3K4me3 occupation at each base pair in the human reference genome (hg19/GRC37) and the *in vivo* coverage of H3K4me3 using ChIP-seq reads.

### 2.6 Identification of TF-binding sites and inter-chromatin interaction sites

The ER-α-bound human chromatin interaction sites were obtained from the ChIA-PET data in hg18 (Fullwood *et al.*, 2009) and transferred to hg19 using liftOver. The AR- and FoxA1-binding sites in prostate cancer cells identified by Wang *et al.* (2009a) and Lupien *et al.* (2008) were transferred to hg19 using liftOver. NANOG-, OCT-, SOX2-, KLF4- and TAF1-binding sites in embryonic stem cells identified by Lister *et al.* (2009) were moved to hg19 using liftOver.

## 3 RESULTS

### 3.1 Sequence specificities of nucleosome modifications

**Fig. 1.**
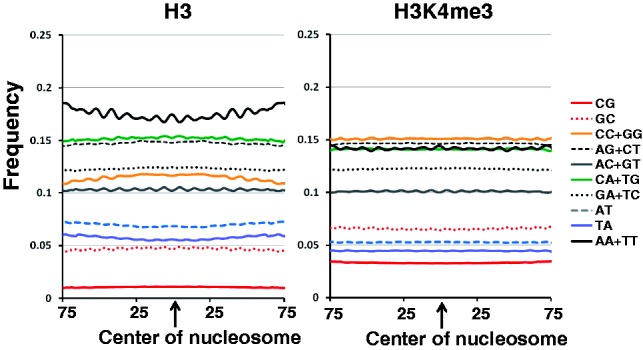
Dinucleotide frequencies in H3 and H3K4me3 nucleosomes. The frequency of every dinucleotide in H3 and H3K4me3 nucleosome-bound genomic sequences was calculated at each position using 3 bp windows. The horizontal axis represents distance from the center of nucleosome-bound sequences

We compared position-specific dinucleotide frequencies between H3 and H3K4me3 nucleosomal DNA sequences. H3K4me3-bound DNA sequences show a 10 bp periodicity of dinucleotide frequencies, but different amplitudes from H3 nucleosome-bound DNA (Supplementary Figs S1 and S2) (Satchwell *et al.*, 1986; Segal *et al.*, 2006). In addition to the 10 bp periodicity, position-specific frequencies of each dinucleotide are within specific discrete ranges and differ from those of H3 nucleosomes (Fig. 1; paired *t*-tests, *P*-values 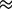 0 for CG frequencies in H3 versus H3K4me3, GC in H3 versus H3K4me3, CC/GG in H3 versus H3K4me3, AG/CT in H3 versus H3K4me3, AC/GT in H3 versus H3K4me3, CA/TG in H3 versus H3K4me3, AT in H3 versus H3K4me3, GA/TC in H3 versus H3K4me3, TA in H3 versus H3K4me3, AA/TT in H3 versus H3K4me3).

**Fig. 2.**
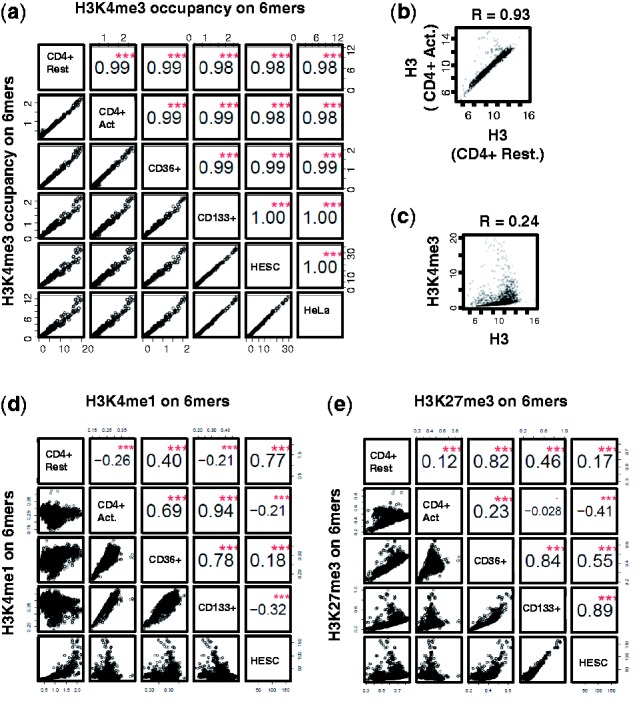
H3K4me3 shows consistent and distinct sequence specificity among various human cell types, whereas other modified nucleosomes do not. (**a–e**) The correlations of the 6mer specificities in H3K4me3 nucleosomes (a), in H3 nucleosomes (b), between H3K4me3 and H3 nucleosomes (c), in H3K4me1 nucleosomes (d) and in H3K27me3 nucleosomes (e). The nucleosome-bound DNAs were identified using the ChIP-seq data from various cell types: CD4 + T cells in resting state (CD4 + Rest), CD4 + T cells in activated state (CD4 + Act), CD36 + erythrocyte stem cell (CD36 + ), CD133 + , HESC and HeLa cells. The values in the squares represent correlation coefficients of 6mer sequence preference between two cell types. The red stars in a square signify that the correlation is highly significant, with the *P*-value close to 0. The vertical and horizontal axes represent the number of ChIP-seq reads covering the 6mer normalized by the number of 6mers in the human genome. Each point represents the enrichment of a 6mer in the ChIP-seq experiments in two cell types. The cell types on the *x*-axis and *y*-axis are marked on the main diagonal

The different dimer sequence frequencies between H3 and H3K4me3 nucleosomes imply that an n-mer (*n* > 2) sequence composition can be a distinct feature of H3K4me3 nucleosomes. We studied 6mer sequences in histone methylations because 6mer sequences are long enough to represent sequence preferences, and the computation is still feasible. The distinct sequence preferences of H3K4me3 and H3 nucleosome are well reflected in 6mer sequence specificities and are consistently found in various cell types (Fig. 2a and b, *r *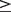 0.98, *P* = 0). In contrast, H3K4me1 and H3K27me3 nucleosomes show no consistent sequence preferences among cell types (Fig. 2d and e). The preferred 6mer DNA sequences of H3K4me3 show only a weak correlation with H3 nucleosome sequence preferences (Fig. 2c, *r* = 0.24, *P* = 0), indicating that H3K4me3 modification uses a mechanism different from that of any other nucleosome.

To examine whether the sequence specificities differ between promoters and non-promoter regions, we calculated sequence specificities of histone modifications in the promoters and non-promoter regions. We found that the sequence specificities of histone modifications are highly significantly correlated between promoters and non-promoter regions (Supplementary Fig. S5), implying that the 6mer sequence specificities of H3K4me3 are consistently maintained across the genome.

### 3.2 A probabilistic model for an H3K4me3 occupation map in the human primary genome sequence

**Fig. 3.**
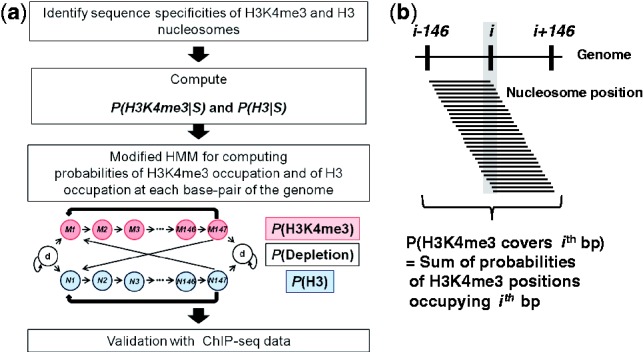
Flow chart to compute the probabilities of H3K4me3 and H3 nucleosome occupation at each base pair in the human genome. (**a**) First, the sequence specificities of H3K4me3 and H3 nucleosomes on 6mer sequences are inferred using *in vivo* ChIP-seq data. Second, the sequence specificities are used to compute the probability that a given sequence S of 147 bp is a potential H3K4me3 nucleosome site and the corresponding probability for an H3 nucleosome site without H3K4me3. Third, a modified fifth order HMM is constructed to compute the probabilities that a given base pair in the human genome is covered by an H3K4me3 nucleosome or an H3 nucleosome. (**b**) The probability of occupation at a base pair is the sum of the occupation probabilities of all the nucleosomes that can occupy the base pair

Using the aforementioned sequence specificities of H3K4me3 and H3 nucleosomes, we can compute the probability, *P*(H3K4me3 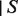), that a given 147 bp or shorter sequence segment (S) is an H3K4me3 nucleosome site, and also *P*(H3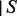) (Fig. 3). In principle, a base pair in the genome sequence can be the *ith* bp of the DNA sequence of a nucleosome (*i* = 1, … , 147). However, at each time at most only one nucleosome can occur at a base position. To consider various possible arrangements of nucleosomes occupying a site, we constructed a modified 5th order (HMM of H3K4me3 and H3 nucleosome occupation (Fig. 3). Our model calculates the probability of every possible arrangement of H3K4me3 and H3 nucleosomes on the whole genome. It distinguishes between H3K4me3 and H3 nucleosomes and integrates all possible arrangements based on forward and backward sequences of every base pair (Fig. 3). Ultimately, our model calculates the sum of the probabilities of all possible H3K4me3 and H3 nucleosomes that can potentially cover the base pair (see Section 2). If the loci associated with a high probability of H3K4me3 nucleosome occupation becomes significantly long, the genomic region may be considered to be fuzzy nucleosome sites as many previous research articles did, including Yuan *et al.* (2005) and Segal *et al.* (2006) (Supplementary Fig. S6).

### 3.3 Association of the genome sequence context and the H3K4me3 occupation

**Fig. 4.**
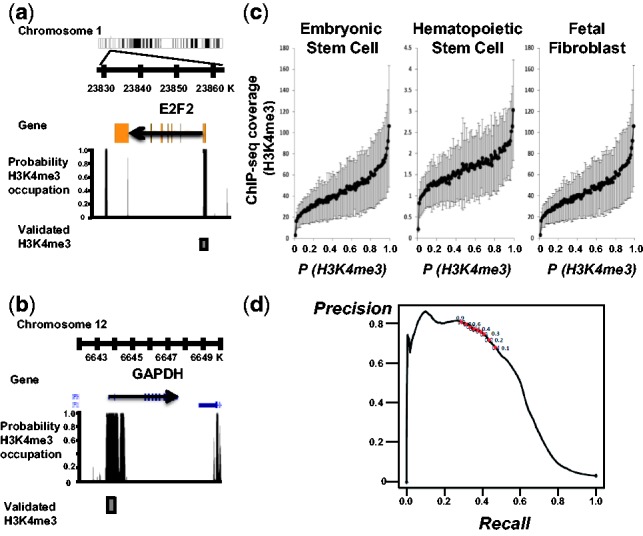
Computed probabilities of H3K4me3 occupation recapitulate *in vivo* occupancies of H3K4me3 across various cell types. (**a** and **b**) The 5′-end of E2F2 and GAPDH genes have been experimentally validated to have a very high level of H3K4me3 using western blot (Steward *et al.*, 2006). Arrow indicates the direction of transcription. The level of black bar is the probability of H3K4me3 occupation at a base pair. A gray box represents the genomic region containing previously validated high-density H3K4me3 nucleosomes. (**c**) H3K4me3 ChIP-seq experimental occupancy is correlated with probability of H3K4me3 nucleosome occupation based on the human genome among cell types. The gray lines represent the 99% confidence interval of the mean ChIP-seq read occupancy. (**d**) Precision-recall analyses of *P*(H3K4me3 occupation). The dots mark cut-off probability of H3K4me3 occupation at 0.1–0.9 increased by 0.1

To examine the association of primary genome sequence context and H3K4me3 distribution, we compared our sequence-based prediction of H3K4me3 occupation of a locus with the occupation level from ChIP-seq experiments. As ChIP-seq experiments cannot provide 1 bp resolution localization of nucleosomes, we use the probabilistic occupation level of H3K4me3 nucleosome and the number of ChIP-seq reads covering the locus. We found several lines of evidence for a significant correlation between our sequence-based prediction of H3K4me3 occupation and the experimental data. First, the 5′-ends of the E2F2 and GAPDH genes have been experimentally shown to carry a high level of H3K4me3 (Steward *et al.*, 2006), and our model indeed predicts that these genomic regions have a high probability of H3K4me3 occupation (E2F2, *r* = 0.73, *n* = 3 × 10^4 ^bp; GAPDH, *r* = 0.76, *n* = 6 × 10^3 ^bp) (Fig. 4a and b). The correlation coefficients of coverage at a base pair resolution are within the ranges observed in biological replications (Leleu *et al.*, 2010).

Second, we compare the probability of a base pair being covered by H3K4me3 and the number of reads covering the base pair in ChIP-seq experiments in various cell types. The probability correlates well with the *in vivo* occupancy of H3K4me3 at each base pair in the 2.5 × 10^9 ^bp mappable genomic region, estimated from ChIP-seq data in diverse cell types, including embryonic stem cells (HESC, *r* = 0.83, *P* = 0, *n* = 2.5 × 10^9 ^bp), fetal fibroblast cells (IMR90, *r* = 0.63, *P* = 0, *n* = 2.5 × 10^9 ^bp), CD133 + hematopoietic stem cells (*r* = 0.55, *P* = 0, *n* = 2.5 × 10^9 ^bp), CD36 + erythrocyte precursors (*r* = 0.54, *P* = 0, *n* = 2.5 × 10^9 ^bp), HeLa (*r* = 0.52, *P* = 0, *n* = 2.5 × 10^9 ^bp) and resting and activated CD4 + T helper cells (*r* = 0.46, *P* = 0, *n* = 2 × 10^9 ^bp) (Fig. 4c). The enrichment of ChIP-seq reads provides probabilistic occupation level as well. In addition, there is possible variation in ChIP-seq experiments and differential regulation of H3K4me3 among cell types (Supplementary Fig. S3). The correlations between our predictions and the *in vivo* data are surprisingly high, suggesting a high accuracy of our model.

Third, we conducted precision/recall analyses to assess the efficiency of our method. The precision level is the proportion of genomic regions occupied by H3K4me3 from the ChIP-seq experiment among the predicted genomic regions at an occupation probability cut-off. The recall level is the proportion of genomic regions predicted to be occupied by an H3K4me3 nucleosome among the genomic regions occupied by H3K4me3 from the ChIP-seq experiment. The experimentally verified positive loci associated with enrichment of H3K4me3 occupation were extracted from the H3K4me3 ChIP-seq experiment in CD4 + T cells (Poisson process, *P* < 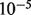). With the *P*(H3K4me3 occupation) 0.5 cut-off, the precision level was 76% and the recall level was 37% in CD4 + T cells (Fig. 4d). As only 5% of the human genome is actually occupied by an H3K4me3 nucleosome in CD4 + T cells, our prediction of H3K4me3 occupation efficiently enriches the true H3K4me3 occupation sites [*χ*^2^ test, H3K4me3 occupation sites in the genome (5%) versus H3K4me3 occupation sites among *P*(H3K4me3 occupation) 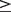 0.5 (76%), *P* = 0]. In view of the fact that only 1% of the genome is predicted with *P*(H3K4me3 occupation) 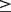 0.5, the enrichment of 37% true positive in *P*(H3K4me3 occupation) is highly significant [*χ*^2^ test, *P*(H3K4me3 occupation) 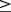
0.5 in total genome (1%) versus *P*(H3K4me3 occupation) 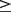
0.5 among true H3K4me3 occupation sites (37%), *P* = 0]. The precision-recall analyses suggest that our method efficiently predicts H3K4me3 nucleosome occupations.

### 3.4 The probability of H3K4me3 occupation at regulator–chromatin interaction sites

**Fig. 5.**
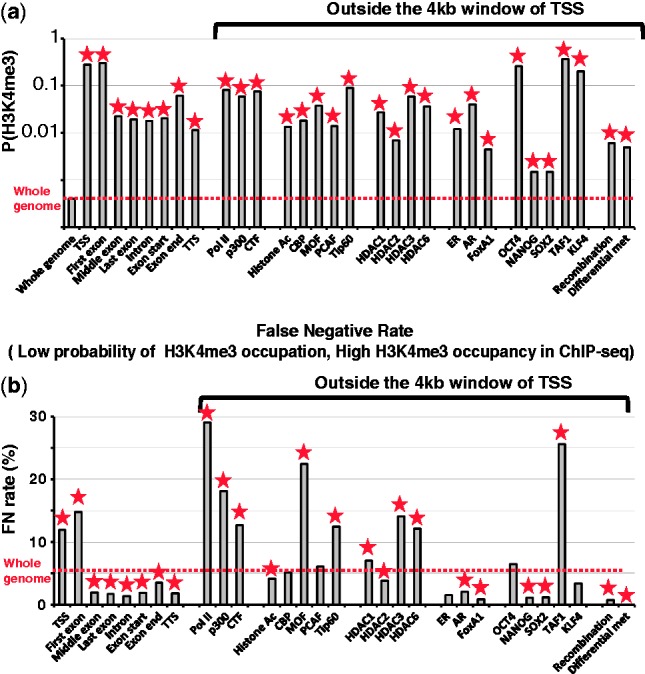
The probability of H3K4me3 occupation on regulatory elements in the human genome. (**a**) The probabilities of H3K4me3 occupation on regulatory elements in the human genome. The red dotted line represents the average probability of H3K4me3 occupation in the whole genome. The vertical axes are in the logarithmic scale. (**b**) The false negative rates at regulatory elements represent the occupation of H3K4me3 not following the high probability of H3K4me3 occupation. Out of the sites with the probability of H3K4me3 occupation <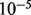, the proportion of sites with the top 0.1% ChIP-seq reads in CD4 + T cells were calculated in a 200 bp window for each regulatory element. Asterisks represent statistical significance, *P* < 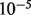

To investigate the role of the predicted H3K4me3 occupation in transcription, we studied its predicted occurrence around a transcribed region. We found a high peak of the probability of H3K4me3 occupation at the TSS and TTS of genes and at the start and end sites of exons (Fig. 5a). To study the probabilistic H3K4me3 occupation profile in higher-order chromatin–chromatin interaction, we examined the relationship between the probability of H3K4me3 occupation and protein factors regulating chromatin structure. Histone acetylases help long-range chromatin interactions between enhancer-bound TFs and the RNA Pol II complex at promoters and enhancers. Insulators are preferentially bound by CTCF and involved in gene regulation and chromatin structural changes. We calculated the read coverage from ChIP-seq of RNA Pol II to estimate the distribution of RNA Pol II on the human genome. The probability of H3K4me3 occupation is significantly correlated with binding of RNA Pol II (Fig. 5a). Using the ChIP-seq data of various histone acetylases, deacetylases and CTCF, we estimated their binding preferences by the number of reads covering a base pair. The histone acetylases included in the analyses are p300, CBP, MOF, PCAF and Tip60, and the histone deacetylases included are HDAC1, HDAC2, HDAC3 and HDAC6. We found that histone acetylases, deacetylases and CTCF preferentially interact with the genome regions showing high probability of H3K4me3 (Fig. 5a). Likewise, the *in vivo* levels of histone acetylations significantly correlate with the probability of H3K4me3 occupation. The significant co-localizations of various histone acetylations on the predicted H3K4me3 sites suggest that the high probability of H3K4me3 occupation represents the sequence context of histone modifications at regulatory elements. Note that p300, a well-known enhancer-binding protein (Visel *et al.*, 2009), preferentially binds the genomic region associated with high probability of H3K4me3 occupation. These observations suggest that the sequence context encoding high probability of H3K4me3 occupation is specifically distributed across the human genome. In particular, the probability of H3K4me3 occupation is correlated with protein factors that affect chromatin structure.

In general, we found many TF-binding sites associated with the high probability of H3K4me3 occupation (Supplementary Tables S1 and S2). The OCT4-, NANOG-, SOX2- and KLF4-binding sites identified by ChIP-seq in HESC (Lister *et al.*, 2009) show high probability of the H3K4me3 occupation. These TFs are key regulators of stem cell identity and closely interact with histone modifications (Boyer *et al.*, 2005). The results suggest that cell-type–specific TFs interact with different patterns of the probability of H3K4me3 occupation.

Finally, we estimated the proportion of H3K4me3 nucleosome occupied sites not following the probability of H3K4me3 occupation. We calculated the false negative rates of the probabilistic occupation map of H3K4me3 at regulatory elements (Fig. 5b). The false negative rate is the proportion of sites showing a high occupancy level of H3K4me3 (a high number of H3K4me3 ChIP-seq reads, Poisson distribution, *P* < 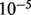) despite a very low probability of the H3K4me3 occupation [*P*(H3K4me3) 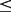
0.00001]. The TSS regions and the first exons show significantly higher false negative rates (12 and 15%) than most other regulatory elements. This suggests that the sequence signature is not only derived from TSS regions and the first exons but also from other genomic regions (Supplementary Fig. S3). Although Pol II-, p300- and CTCF-binding sites contain a high probability of the H3K4me3 occupation, they still show high false negative rates. This suggests that some H3K4me3 occupation in these regions is determined not only by sequence context but also by epigenetic factors not explained by DNA sequence context.

## 4 DISCUSSION

The different sequence preferences of H3K4me3 and H3 nucleosomes imply distinct mechanisms in their arrangement on the genome. Although the H3 nucleosome is mainly distributed by the bending property of DNA sequences (Segal *et al.*, 2006; Vafabakhsh and Ha, 2012), the H3K4me3 nucleosome is regulated by protein factors recognizing specific sequence compositions, such as non-methylated CG-rich sequence signatures. The sequence-specific protein factors interact with histone methylase complexes specific to H3K4me3, such as Setd1. In mouse, insertion of non-methylated CpG-rich sequences leads to a high density of H3K4me3 occupation. For example, Cfp1 specifically binds to non-methylated CpG rich sequences and recruits the Setd1 complex (Thomson *et al.*, 2010). In mouse and human, PRDM9, a meiosis-specific H3K4me3 histone methyltransferase, recognizes the target sites by the sequence specificity and deposits H3K4me3 nucleosomes at meiosis recombination hotspots (Baudat *et al.*, 2010). Therefore, the sequence context showing high probability of H3K4me3 occupation at important regulatory elements across the genome may attract chromatin modifying factors.

We found consistent and distinct sequence specificity only for H3K4me3 but not for other histone modifications. It is possible that other histone modifications are associated with some sequence specificities that cannot be detected by the 6mer sequence preferences. Another possibility is that sequence-independent epigenetic factors regulate other histone modifications. Indeed, recently Tsai *et al.* (2010) showed that long non-coding RNAs guide the H3K27me3 enrichment, although the sequence specificity of the non-coding RNAs has not yet been identified.

Previously, Yuan *et al.* (2005) also used HMM to estimate nucleosome occupation probabilities from microarray data of nuclease-treated DNA. Their hidden state was the occupation of nucleosome, and the observations were microarray Cy3 intensities. Their emission probabilities were Cy3 intensities compared with the input DNA signal, Cy5. They characterized nucleosome location based on the consecutive eight probe intensities. On the other hand, our purpose is to compute the occupation probabilities of different types of nucleosomes from the genomic sequence alone. We first showed that the distinctive sequence specificities of H3K4me3 and H3 nucleosomes are consistent among different cell types. We then used the observed sequence specificities of H3 and H3K4me3 to predict their occupation probabilities at each mappable base pair from the genomic sequence alone. In our HMM model, the hidden state is the occupation of H3K4me3 (or H3) nucleosome at a base pair, and the observed state is the genome sequence. The emission probabilities in our model are sequence specificities of H4K4me3 (or H3). The predicted probabilities of H3K4me3 occupation are consistent with experimental data from various human cell types. Although ChIP-seq data contain noise, the probability of H3K4me3 occupying a genome region can be estimated from the sequence reads, as long as the number of reads is large enough for the statistics to be meaningful.

Our model for predicting an H3K4me3 nucleosome map from the human genome sequence is similar to Segal *et al.*’s model (2006) in that we assume that nucleosome formation in a genome is a dynamic process, and we calculate the nucleosome occupation probability at a locus considering all possible nucleosomes that can potentially cover the site and their forward and backward configurations in a genome. However, our model differs from theirs in at least three aspects. First, our model incorporates the specific modification status of each nucleosome and distinguishes between H3 and H3K4me3 occupations. On the other hand, Segal *et al.* and other studies did not distinguish between H3 and H3K4me3 nucleosomes, but considered general nucleosomes, i.e. micrococal nuclease-sensitive regions. Second, we compute the sequence compositions of DNA segments in H3 nucleosomes and in H3K4me3 nucleosomes separately, whereas Segal *et al.* computed the composition in extracted DNA segments in all forms of nucleosomes after treatment of micrococcal nuclease. Third, we aim to infer the probabilistic occupation level of the H3K4me3 nucleosome based on the genome sequence context, whereas Segal *et al.* computed the probabilistic positioning of a base pair within 147 bp nucleosomal DNAs. Our model is based on the ChIP-seq experiments. ChIP-seq enables us to infer the probable coverage of a locus but not exact positions within nucleosomes. Therefore, our model can only estimate the probabilistic occupation levels of H3K4me3 and H3 nucleosomes based on the whole-genome sequence context.

In conclusion, our results underscore a relationship between genome sequence and chromatin remodeling. In addition, sequence-independent epigenetic regulation of nucleosomes also contributes to chromatin modifications. Our analyses of the H3K4me3 sequence signature will be a valuable starting point for future studies of the genome structure. Ultimately, the computational approach may help identify epigenetic and genetic factors that regulate the chromatin structure.
